# Green extraction and application of yellow natural curcumin colorant from *Curcuma aromatica* rhizomes for silk dyeing

**DOI:** 10.1038/s41598-024-63927-7

**Published:** 2024-06-06

**Authors:** Noman Habib, Fatima Batool, Shahid Adeel, Muhammad Naveed, Aamir Ali, Rony Mia, Mohammed A. Assiri

**Affiliations:** 1 Department of Botany, Government College University Faisalabad, Faisalabad, 38000 Pakistan; 2 Department of Botany, Division of Science and Technology, University of Education Lahore, Lahore, 54770 Pakistan; 3 Department of Applied Chemistry, Government College University Faisalabad, Faisalabad, 38000 Pakistan; 4 Center for Global Health Research, Department of Medical Biotechnology, Saveetha Medical College and Hospital, Saveetha Institute of Medical and Technical Sciences (SIMATS), Tamil Nadu, 602105 India; 5 Department of Chemistry, Faculty of Science, King Khalid University, P.O. Box 9004, 61413 Abha, Saudi Arabia

**Keywords:** *Curcuma aromatica*, Silk, Acacia, Mordanting, Pomegranate, Microwave, Environmental monitoring, Pollution remediation

## Abstract

Green products such as plant tints are becoming more and more well-known worldwide due to their superior biological and ayurvedic properties. In this work, colorant from Amba Haldi (*Curcuma aromatica*) was isolated using microwave (MW), and bio-mordants were added to produce colorfast shades. Response surface methodology was used to develop a central composite design (CCD), which maximizes coloring variables statistically. The findings from 32 series of experiments show that excellent color depth (K/S = 12.595) was established onto MW-treated silk fabric (RS = 4 min) by employing 65 mL of radiated aqueous extract (RE = 4 min) of 5 pH cutting-edge the existence of 1.5 g/100 mL used sodium chloride at 75 °C for 45 min. It was discovered that acacia (keekar) extract (1%), pomegranate extract (2%), and pistachio extract (1.5%) were present before coloring by the use of bio-mordants. On the other hand, upon dyeing, acacia extract (1.5%), pomegranate extract (1.5%), and pistachio extract (2%) have all shown extremely strong colorfast colors. Comparatively, before dyeing, salts of Al^3+^ (1.5%), Fe^2+^ (2%), and TA (1.5%) gave good results; after dyeing, salts of Al^3+^ (1%) and Fe^2+^ (1.5%) and TA (2%) gave good results. When applied to silk fabric, MW radiation has increased the production of dyes recovered from rhizomes. Additionally, the right amount of chemical and biological mordants have been added, resulting in color fastness ratings ranging from outstanding to good. Therefore, the natural color extracted from Amba Haldi can be a sustainable option for the dyeing of silk fabric in the textile dyeing and finishing industries.

## Introduction

Green products like natural dyes are now an alternative to synthetic colorants in all fields^1^. The daily production, formulation, and finishing operations for synthetic colors use mutagenic and carcinogenic components, which are dangerous in destroying the globe's beauty^2,3^. In addition to water bodies, these bodies' pH, COD, and BOD levels are becoming unbalanced due to such effluents, which will also destroy aquatic life and the beauty of lakes and rivers^4^. Industrial wastes also distress worldwide health by affecting allergic reactions and hostile skin situations^5^. By repelling hazardous waste elements, including various compounds like dyes, surfactants, and heavy metal ions, synthetic dyes are one of the causes of increasing environmental pollution and upsetting the eco-balance^6^. People demand health-supporting and eco-friendly products such as natural dyes. Natural dyes are derived from plants, insects, animals, algae, lichens, fungi, bacteria, minerals, and rocks^7,8^. The researchers appreciate natural dye sources because they are safe for the environment, nontoxic, renewable, non-hazardous^9^, biodegradable, and non-carcinogenic^10^. Due to their bacterial resistance, UV-protective properties, and antioxidant properties, many natural colorings are gaining recognition for their diverse applications in various fields^11^. These coloring agents produce more appealing, gentler, and calming colors^12^. These qualities repel insects, eliminate odors, and resist burning^13^. Natural dyes face two main issues: low yield and poor shade.

Some classic methods are used to improve extractions using reduced energy, and solvent, however promising results still need to be achieved. In the textile industry, a wide variety of extraction technologies, such as ultrasonic waves, gamma radiation, and microwave (MW) radiation, are utilized widely on a global scale^14^. The purpose of these technologies is to improve the extraction and usage of natural colorants. On the other hand, it is essential to take into consideration the potential complications that are associated with gamma radiation and ultrasonic waves in comparison to MW radiation. When it comes to the extraction of natural dyes, gamma radiation presents challenges due to its potential for molecular modification and the production of free radicals^15^. Ultrasonic waves, on the other hand, can occasionally cause heating effects, which might possibly lead to the deterioration of sensitive molecules^16^. When it comes to the process of natural dyeing, the employment of microwaves has become increasingly popular owing to the fact that they are both cost-effective and capable of saving time^17,18^. This method makes it possible to create favorable solvent-powder interactions in a consistent heating source. MW leads to outstanding energy transfer and the possibility for large dyeing revenues in textiles^19^. The ray uniformly acts upon the system, extracting required material through outstanding mass transfer kinetics by using less energy, time and solvents^20^. In addition, materials absorb microwaves, however very low-energy particles, or photons, are left behind. The atomic and molecular structures are too strong for this energy to disintegrate them. Thus, the MW energy does not damage or undermine the structure of the textile materials^21^. This is due to molecular diffusion is more efficient when materials are heated using a microwave, it has become possible to dye textiles uniformly and quickly^22^.

In natural coloring, mordants are used to improve color and fastness characteristics. Two types of mordants are used in natural dyeing: chemical and bio-mordants. By creating interactions between the fibers, mordant, and dye, mordants are crucial when dyeing with many natural dyes^23^. Many metal salts are used, however, Cu, Co, and Cr electrolytes are toxic and should not be used^24,25^. These harmful compounds produce a wide range of hues when coupled with natural dyeing and fabric coloring, however, their effluent after use harms aquatic and human life^26^. Mordanting's tendency has shifted from chemical to bio-mordants in the natural coloring process, making the process cleaner, greener, and pollution-free^27^. These mordants can substitute metal salts for textiles' ecological coloring and create new color gamutes^28^.

**Figure 1:**
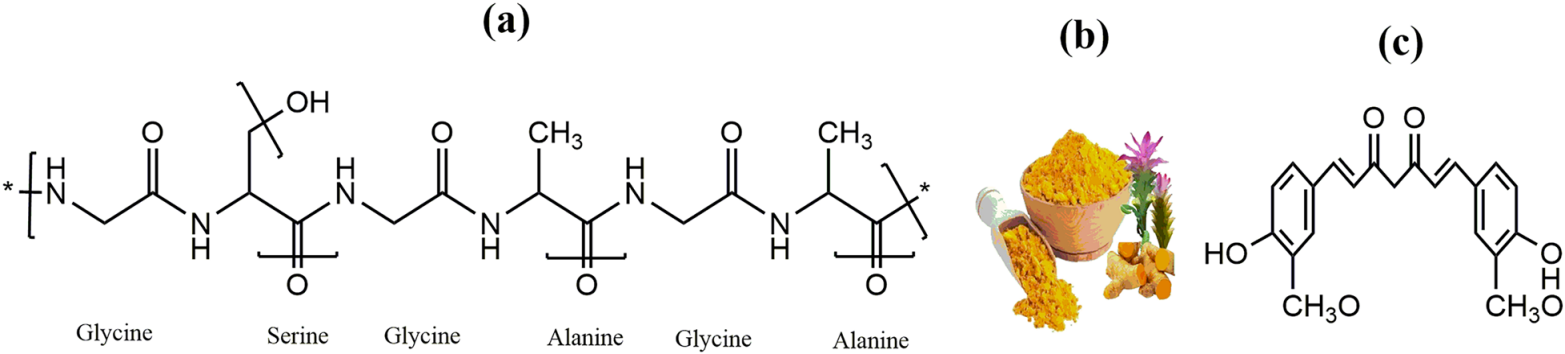
(**a**) Structure of silk. (**b**) Amba Haldi. (**c**) Structure of curcumin.

This study explains a natural crop of the Zingiberaceae family called Amba Haldi (*Curcuma aromatica*) for silk dyeing (Fig. 1). Amba Haldi, also known as turmeric, has excellent biological characteristics such as anti-bacterial, antifungal, and antioxidant^29^. It is a perennial or evergreen herb. India and Indonesia are the countries of origin for this plant, although it has also been cultivated in Europe and the United States^30^. With properties that include anti-inflammatory, wound healing, melanogenic-resisted, and free radical rummaging, as well as tumor-resisted, cancer-resisted, anti-repellent, antitussive, anti-platelet, and nephrotoxic-resistant activity, this rhizome is a marvel of medicine^31^. This rhizome has curcumin, which impacts the yellow color of a matrix^32^. Therefore, the yellow color was obtained from the natural dye extracted from the Amba Haldi extractions. The objective of the research is shown below.

1. To improve extraction of colorants from Amba Haldi under the effect of MW radiation.
2. Optimize dyeing variables through Response Surface Methodology (RSM) Design using optimum extraction and irradiation conditions.
3. To observe the physiochemical environment of silk and silk fabric through SEM and FTIR tools after irradiation.
4. To improve colorfastness by utilizing eco-friendly chemicals and bio-mordants under optimum conditions.

## Material and methods

### Collection of materials

Amba Haldi (wild turmeric) is a cradle of natural yellow coloring. Acacia bark powder (*Acacia nilotica*), pomegranate rinds (*Punicagranatum*), and pistachios *(Pistaciavera* L.) are used as sources of bio-mordants. In contrast, Al, Fe, and Tannic, used as sources of chemical mordants, were procured from the local market in Faisalabad, Pakistan. All plant cradles were cleaned with distilled water and then dried under shade. The plant source was passed through a filter with a mesh size of 20 to obtain a powder with uniformly sized particles. The silk fabric with a GSM of 32 was washed with neutral soap at 80 °C for 30 min to formulate for coloring.

### Irradiation and dyeing process

To isolate colorants, fine plant materials were boiled in aqueous, acidic, and basic solutions for 45 min., keeping Material: Liquor (M:L) of 1:25. Irradiated and unirradiated extracts were used to dye silk fabric using three different types of medium, including aqueous acidic and basic solutions. The fabric and extracts were microwaved for 10 min at 2 min intervals. The extract-to-fabric ratio was kept at 1:25 and radiated and unradiated extracts were used to dye both treated and untreated fabrics at 80 °C for 45 min.

### Optimization of coloring and mordanting surroundings

**Figure 2:**
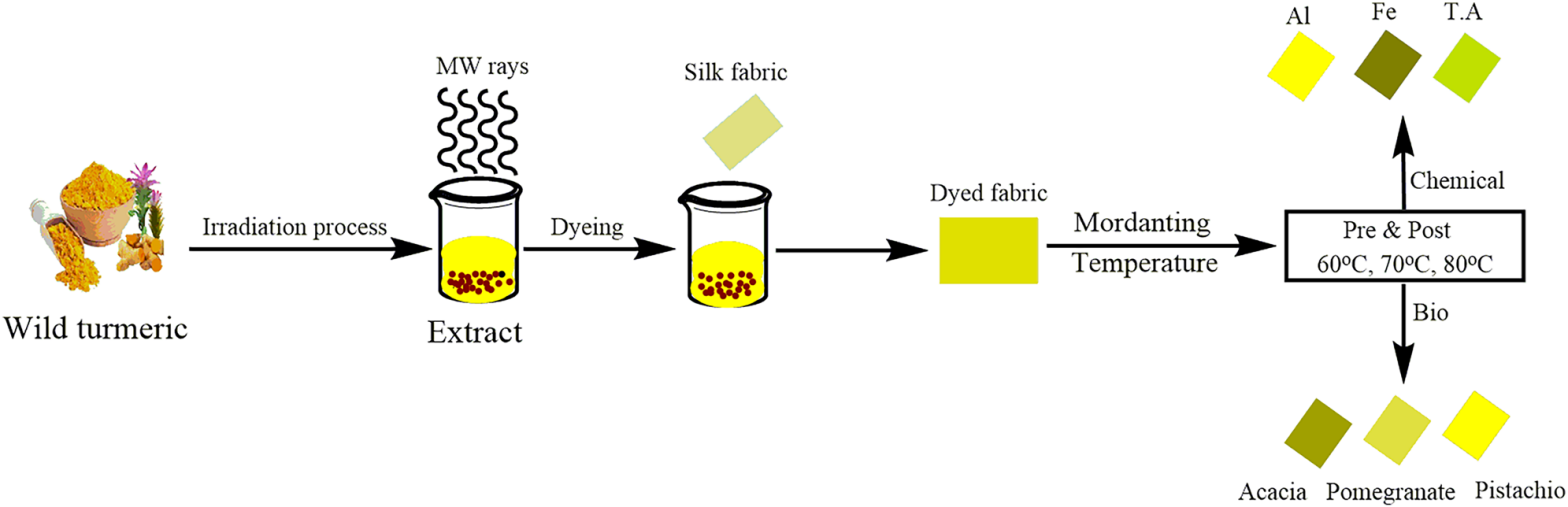
Schematic presentation of silk fabric dyeing using wild turmeric dyes.

Many coloring parameters, containing temperature, time, pH, and extract volume, were statistically optimized using response surface methodology. In 32 trials utilizing the statistical model, dyeing was done at 75–80 °C for 45 min. I used 135–220 ml of 5–7 pH extracts with 2 g/100 mL of salt as an exhausting agent. Before and after-mordanting were done at 75–80 °C for 45 min. to increase the color intensity and colorfastness of fabric dyed below perfect conditions; for this purpose, chemical mordants and (1 g/100 mL) of salt of Al, Fe, and TA and 0.5–2.5 ml of bio and and in comparison 0.5, 2.5 g/100 mL of extract of bio mordants such as acacia bark powder (*Acacia nilotica*), pomegranate rinds (*Punica granatum*) and pistachio (*Pistacia vera*) were employed at 75–80 °C for 45 min. It observed an anchors-to-fabric ratio of 25:1 pre and post-coloring. The illustration of the work is shown in Fig. 2.

### Evaluation of color characteristics of dyed fabrics

Finally, all dyed fabrics' color characteristics (K/S, L*, a* & b*) were examined through a Colori-spectrophotometer (CS = 410) at the Department of Applied Chemistry Government College University Faisalabad, Pakistan. The Noor Fatima Fabrics (Private) Quality Control Laboratory in Faisalabad, Pakistan, was rated the colorfastness attributes of the best mordanted colored fabrics using ISO typical techniques of ISO 105 B02 (2014) for light, ISO 105-CO3 A2S (2013) for washing, and ISO 105 X12 (2002) for rubbing.

## Results and discussion

**Figure 3:**
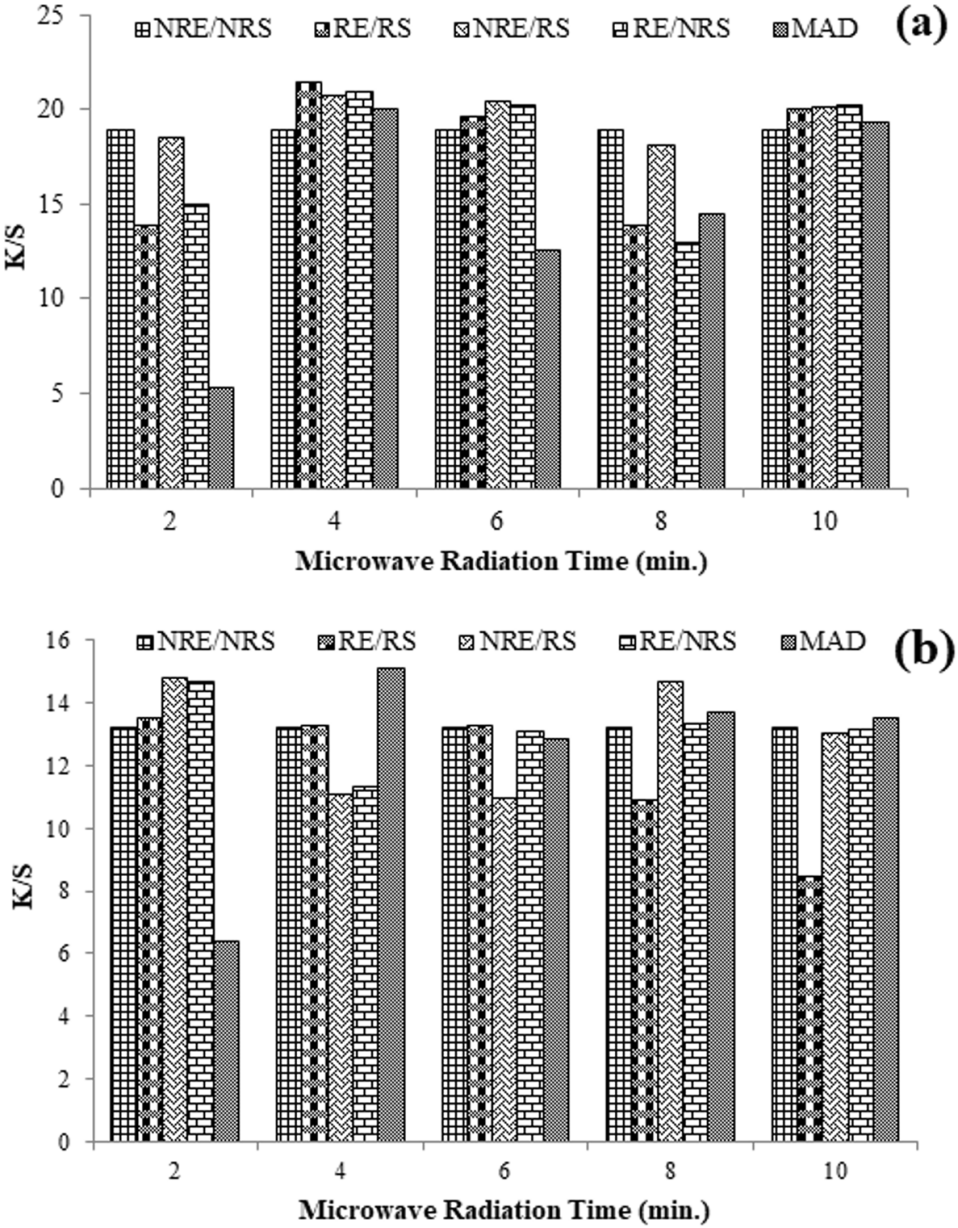
Using MW radiations before and after aqueous (**a**) and acidic (**b**) medium used Amba haldi extract dyeing silk fabric (here, NRE/NRS = non-radiated extract/non-radiated silk, RE/RS = radiated extract/radiated silk, NRE/RS = non-radiated extract/radiated silk, RE/NRS = radiated extract/non-radiated silk, and MAD = microwave assisted dye).

Natural products employ microwaves because of their level, consistent, and energy-efficient characteristics^33^. MW rays change the efficient molecules at a specific level by breaking their boundaries, depriving them of changing the biological nature of bio-potent molecules^34^. MW rays exertion via the powder-solid appliance, which incomes lower solvent for the collaboration of dyeing, promising to improve energy transfer^35,36^. If a low amount (duration) of radiation is applied to an isolation system, the rays will not be able to crack the cell wall, making this good yield impossible. Similarly, the level (time) of rays also interrupts the real purpose of the dyeing since, laterally, with potential isolation, other molecules also progress, affecting the actual yield during application. A similar condition has been experienced in our research. The consequences disclose that the water-made extract (aqueous extract) has given excellent color depth before MW rays upon irradiation for up to 4 min. Applying heated extract has given extracted yield (K/S = 21.392) onto treated silk (Fig. 3).

**Figure 4:**
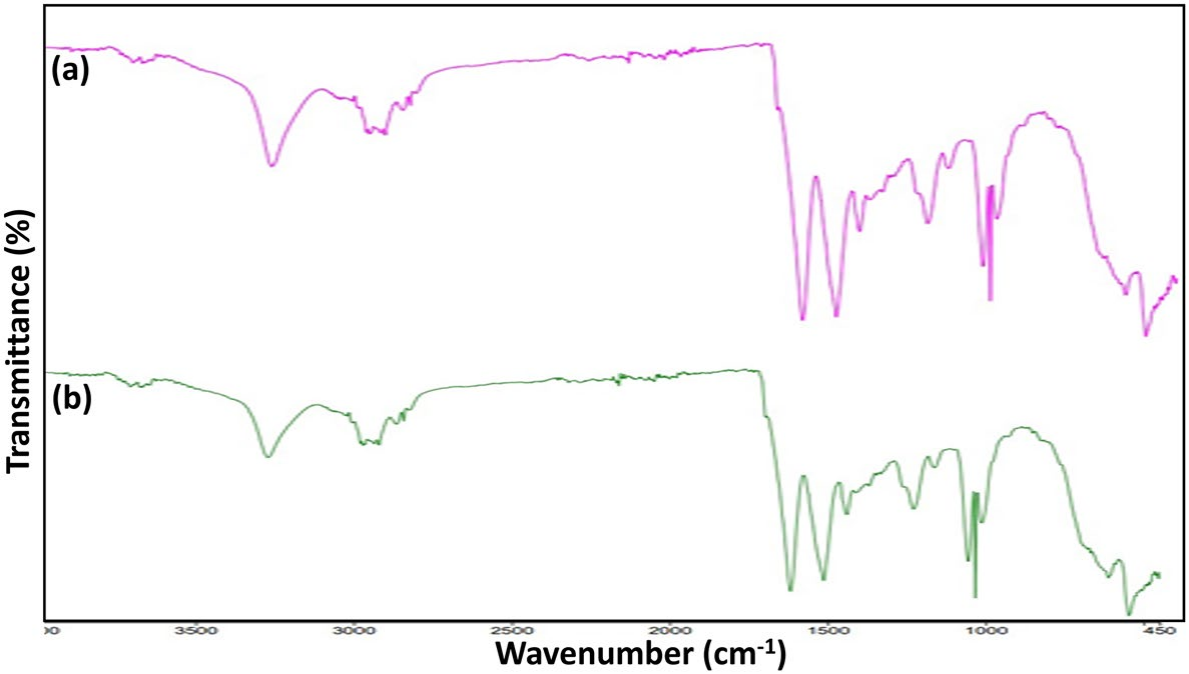
Spectral view of functional groups in Amba Haldi extract before (a) and after (b) MW radiation.

Similarly, under MAD conditions, the treatment of fabric and extracts produced low yields for up to 2 min (K/S = 0.2126). This could be because other molecules isolated with the curcumin also contributed to the dyeing process, changing the color of the colorfastness. The color coordinates presented in Table 1 show that before the treatment of extract and fabric (NRE/NRF), the shade is brighter (L* = 72.50) in the presence and reddish yellowish and darker in the shade (a* = 6.61, b* = 85.62). After 4 min of exposure, the shade brightened and became reddish yellow (a* = 4.61; b* = 91.17). The shade power was low (K/S = 13.209) with a darker shade tone before treating the materials and extract with an acidic medium. However, after 4 min. of fabric irradiation utilizing treated extract and fabric, the hue became less reddish yellow. However, using a dark tone (L* = 74.58) that had a darker hue (a* = − 0.29; b* = 79.91), the shadow strength was marginally improved (K/S = 15.106). As a result, the separation of curcumin from Amba Haldi and the ensuing silk dyeing were not supported in an acidic media. The other characteristic is the surface tuning of silk materials (Fig. 4).

**Table 1:** Color coordinates of aqueous and acidic medium before and after microwave radiation.

Medium	Time (min)	Sample code	*K/S*	L*	a*	b*
Aqueous	Control	NRE/NRS	18.917	72.50	6.61	85.62
	4 min	RE/RS	21.392	75.39	4.61	91.17
Acid	Control	NRE/NRS	13.209	71.70	2.76	73.71
	4 min	MAD	15.106	74.58	− 0.29	79.91

L*: lighter (value increase)/darker (value decrease); a*: redder (value increase)/greener (value decrease); b*: yellower (value increase)/bluer (value decrease).

**Figure 5:**
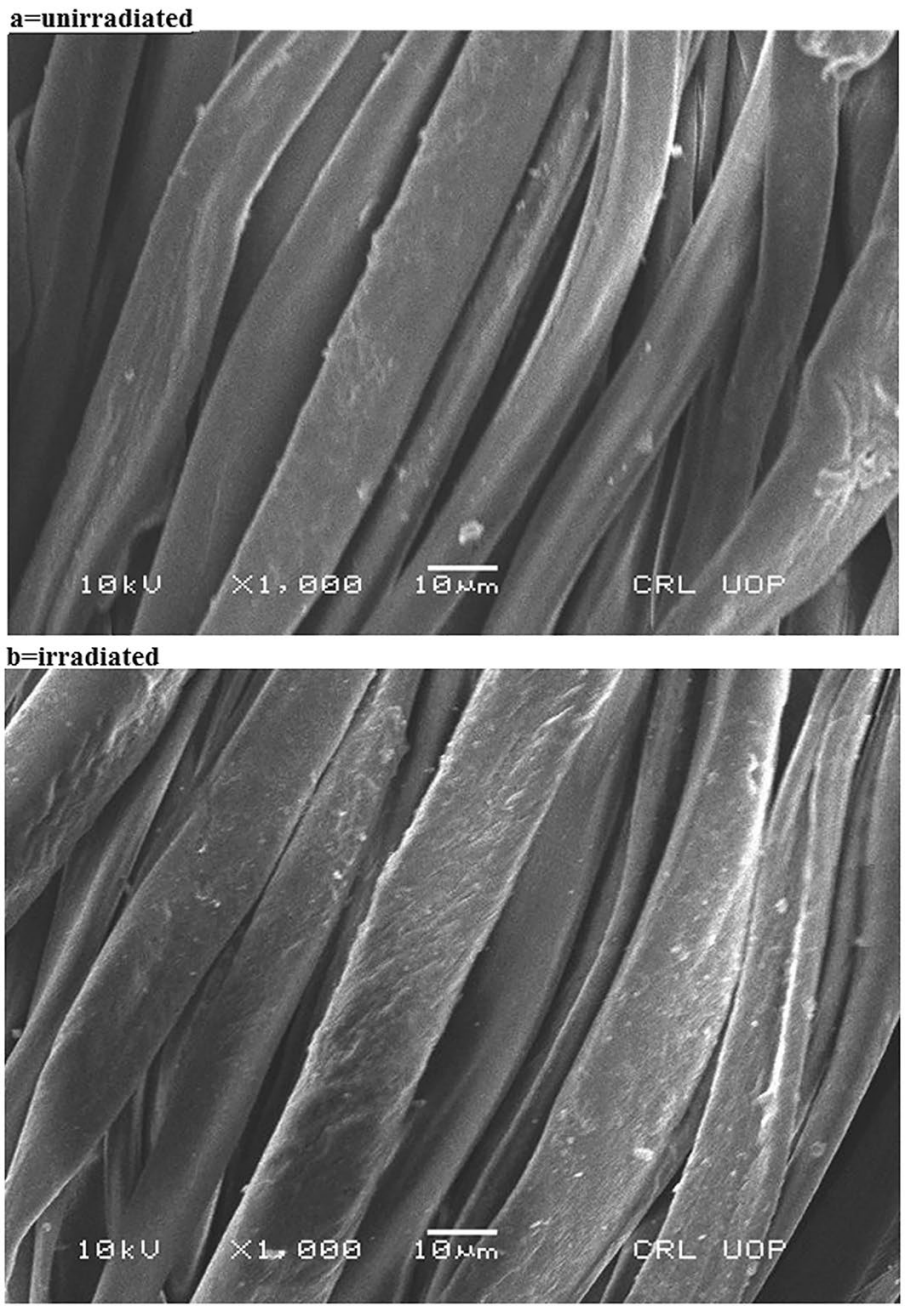
Scanning electron microscope images of silk fabric before (**a**) and after (**b**) under MW radiation.

Previously, our researchers and others found that the MW rays scratch the fabric surface, which helps increase its ambient application. The images perceived under a scanning electron microscope show that the silk fiber was peeled after irradiation, giving encouraging results after dyeing (Fig. 5). MW rays have thereby physically altered the superficial. SEM images were taken before and after radiation for up to 4 min. In Fig. 4 demonstrate that the microwave treatment did not alter the stretching peak of –OH (3300 cm^−1^), –CH group (1200 cm^−1^), and C=O peak at 1075 cm^−1^. This fact demonstrated that the substrate's (silk fabric) chemical makeup is unaffected by microwave radiation. This physiochemical analysis demonstrates that MW rays enhance silk's color when dyed with curcumin in an Amba Haldi aqueous extract. It is strongly advised that the aqueous extract used to dye silk fabrics be MW processed for a maximum of 4 min to achieve isolation. Because low or high levels consistently yield positive outcomes, the dyeing parameter in silk dyeing always produces an auspicious tint.

The coloring parameters always add value to proteinous fabric bio-dyeing at fixed conditions. The designed mode was run individually; the apparent results revealed that if irradiated silk is dyed for 45 min at 65 °C with 65 mL, irradiated extract of 5 pH having 1.5 g/100 mL of salt 65 mL of excellent yield is obtained (Table 2). Extracting pH is essential because the result of colorants plays its role in giving the desired stable shade. Curcumin from Amba Haldi is very sensitive to pH, whereas its application to silk needs acidic pH. Hence, for firm shading, the extract of 5 pH has been optimized.

**Table 2:** Color strength of silk fabric colored with Amba haldi isolation under various conditions employed Response Surface Methodology.

Exp. no	pH	volume	Time	Temperature	salt (g/100 ml)	K/S
1	4	35	35	85	1	3.8171
2	6	35	35	65	1	6.2819
3	4	55	35	65	1	6.4117
4	6	55	35	85	1	6.6181
5	4	35	35	65	2	3.8418
6	6	35	35	85	2	4.4403
7	4	55	35	85	2	6.3383
8	6	55	35	65	2	6.5247
9	4	35	55	65	1	3.9142
10	6	35	55	85	1	4.4403
11	4	55	55	85	1	4.8049
12	6	55	55	65	1	2.5998
13	4	35	55	85	2	3.9920
14	6	35	55	65	2	4.4176
15	4	55	55	65	2	5.9039
16	6	55	55	85	2	4.6481
17	3	45	45	75	1.5	2.6250
18	7	45	45	75	1.5	5.3147
19	5	25	45	75	1.5	3.5369
20	5	65	45	75	1.5	12.595
21	5	45	45	75	0.5	6.3921
22	5	45	45	75	2.5	7.7284
23	5	45	25	75	1.5	5.6882
24	5	45	65	75	1.5	5.5510
25	5	45	45	55	1.5	3.3314
26	5	45	45	95	1.5	5.4411
27	5	45	45	75	1.5	6.3089
28	5	45	45	75	1.5	6.2342
29	5	45	45	75	1.5	6.0473
30	5	45	45	75	1.5	5.9392
31	5	45	45	75	1.5	9.2431
32	5	45	45	75	1.5	13.081

Similarly, extract value is also essential as a low extract amount gives low strength, whereas high-value extract may add value in sorbing colorant as aggregates. This cluster gathers to find difficulty in sorbing into the surface-modified silk voids and remains on the surface on washing, stripped to give low yield. Hence, 65 mL of Amba haldi extract with a pH of 5 is suitable for high yield. Salts also play a role in exhausting the colorant from the dye bath towards the fabric. This process can be achieved if the selected amount of salt solution 1.5 g/100 mL is added occasionally till the end of dyeing. Contact levels, whether heating the dye bath (75 °C) or 45 min (Table 3). It also has a promising role because less heating cannot accelerate dye molecules towards fabric and heating level, and its contact can also favor the desorption of colorant. Less colorant is adsorbed between cases, and low color yield is found. Hence, MW ray treatment has reduced the salt amount and contact time of colorant with fabric, heating, and exhaust value.

**Table 3:** Statistical analysis of 2-way ANOVA using Amba haldi extract dyed silk fabric.

Source	DF	Adj SS	Adj MS	F-value	P-value
Model	15	0.483821	0.032255	11.03	0.000
Linear	5	0.058328	0.011666	3.99	0.021
pH	1	0.032363	0.032363	11.07	0.005
Volume	1	0.001814	0.001814	0.62	0.045
Salt	1	0.002755	0.002755	0.94	0.349
Time	1	0.019337	0.019337	6.62	0.023
Temperature	1	0.10652	0.010652	3.64	0.079
Square	2	0.326385	0.163192	55.83	0.000
pH*pH	1	0.291723	0.291723	99.80	0.000
Volume*volume	1	0.014796	0.014796	5.06	0.042
2-Way interaction	8	0.134643	0.016830	5.76	0.003
pH*salt	1	0.088213	0.088213	30.18	0.000
pH*temperature	1	0.002734	0.002734	0.94	0.351
Volume*salt	1	0.030041	0.030041	10.28	0.007
Volume*time	1	0.015640	0.015640	5.35	0.038
Volume*temperature	1	0.011632	0.011632	3.98	0.067
Salt*time	1	0.052043	0.052043	17.80	0.001
Salt*temperature	1	0.001090	0.001090	0.37	0.552
Time*temperature	1	0.009332	0.009332	3.19	0.097
Error	13	0.037999	0.002923	–	–
Lack-of-fit	10	0.025060	0.002506	0.58	0.774
Pure error	3	0.12939	0.004313	–	–
Total	28	0.521820	–	–	–

**Figure 6:**
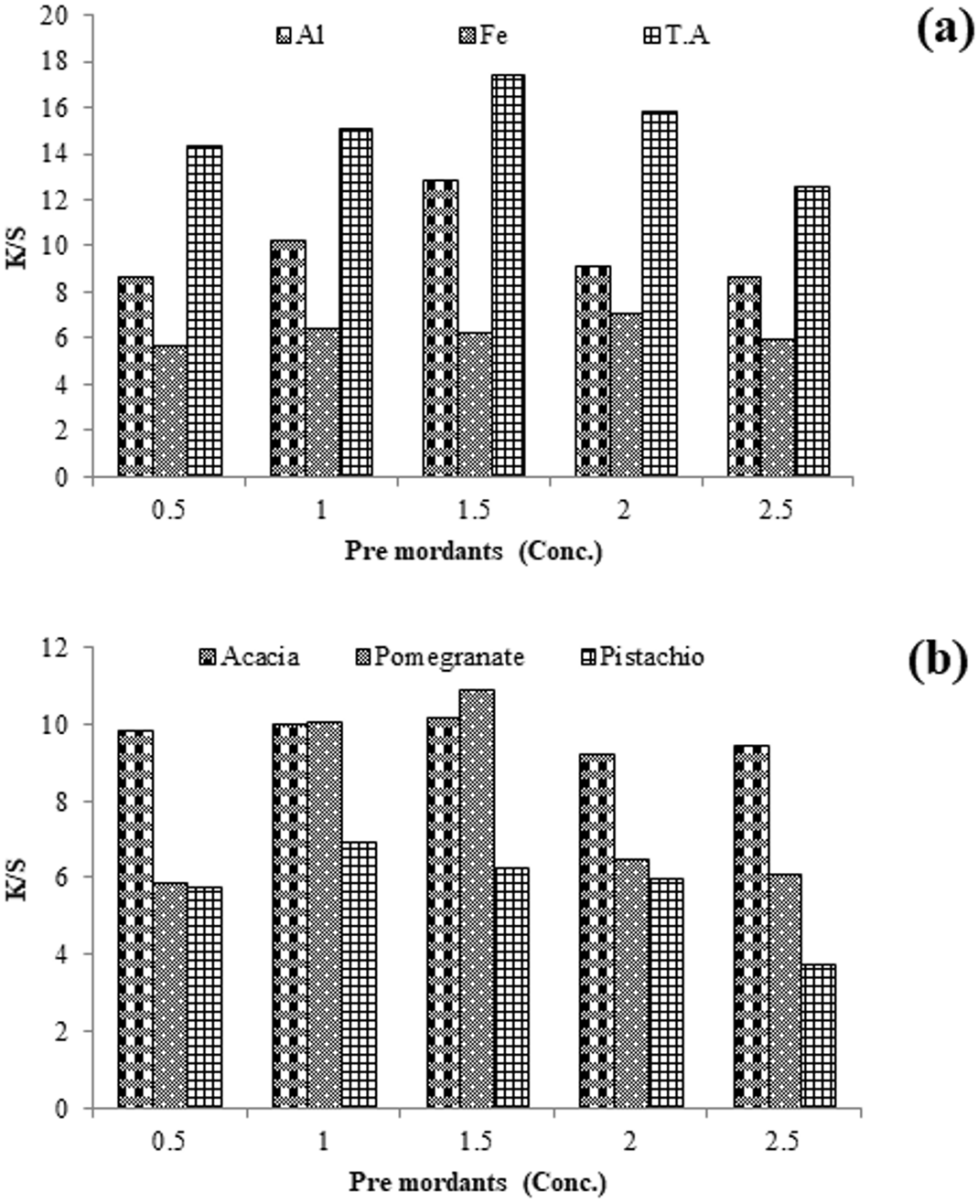
Chemical anchors (**a**) and bio-anchors (**b**) pre-coloring of silk with Amba Haldi extracts under microwave radiation.

**Figure 7:**
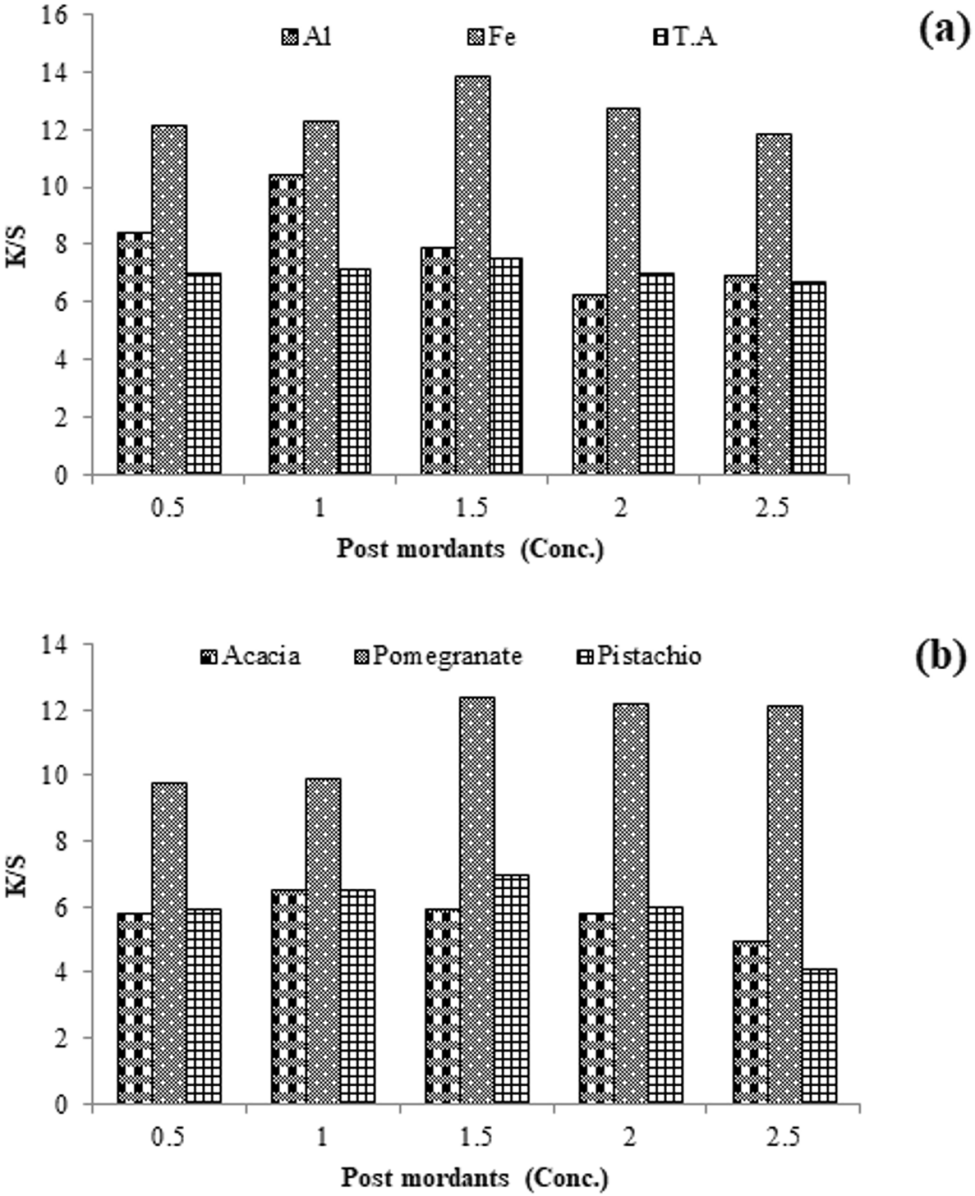
Chemical anchors (**a**) and bio-anchors (**b**) post-coloring silk with Amba haldi extracts under microwave radiation.

Mordanting is a complex forming technique onto fabric to overcome the limitation of poor colorfastness of natural colorant. This is done before or after dyeing using the selected mordant solution per fabric height. The results given in Fig. 6 revealed that 1.5 g/100 mL of Al salt before dyeing and 1 g/100 mL of Al salt solution have developed firm, brightened shades. Similarly, 2 g/100 mL of iron salt before dyeing and 1.5 g/100 mL of Fe salt after dyeing have given firm darker shades. It uses 1.5 g/100 mL of tannic acid in its solution before coloring and 2 g/100 mL after dyeing, giving firmer, darker shades. Preferably, before coloring, 1.5 g/100 mL of tannic acid and 1.5 g/100 mL of iron salt after dyeing is optimum to get a stable shade on silk using Amba Haldi extract (Fig. 7).

**Figure 8:**
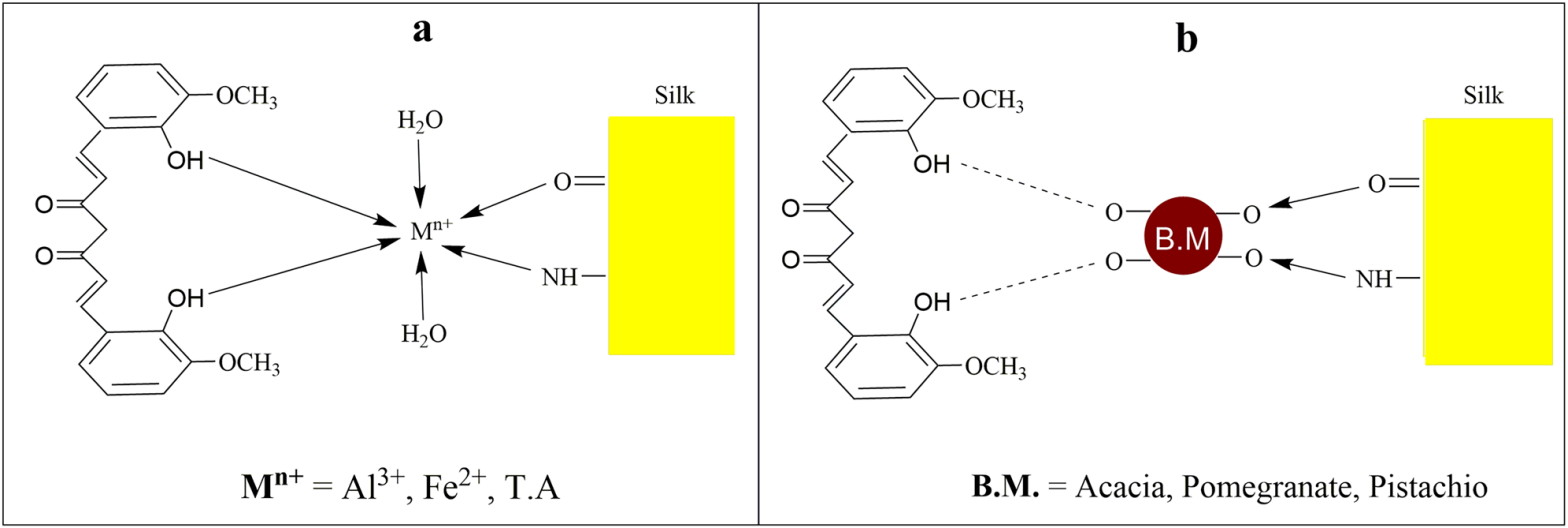
The metal dye interaction of chemical anchors (**a**) and bio-anchors (**b**) silk fabric with Amba haldi extract.

Metal salts play an important role in this process by exploiting their metal sites (Al^3+^, Fe^2+^ and Tannic Acid) to engage with the –OH groups of the colorants and the amido units of silk. This interaction results in the formation of coordinating covalent bonds, which contribute to the generation of colors that are stable and long-lasting^37^. The interaction that is envisaged to take place between metal salts and the dye and the silk fabric is shown in Fig. 8a. Using bio-mordants is an alternative method to replace toxic chemicals. Because bio-sources are mostly present in the Greek, Indian, and Chinese systems of medicine and exhibit wonderful medicinal properties, these bioactive molecules give soothing and colorfast shades when interacting with fabric and dye molecules via H-bonding (Fig. 8)^38^. In this study, acacia, pistachio, and pomegranate have been used before and after dyeing. The results reveal 1 g/100 mL of acacia extract, 1.5 g/100 mL of pistachio extract, and 2 g/100 mL of pomegranate extract before dyeing silk with Amba haldi extract. Similarly, 1.5 g/100 mL of acacia extract, 2 g/100 mL of pistachio extract, and 1.5 g/100 mL of pomegranate extract after silk dyeing with Amba haldi has given stable shades. Comparatively, pomegranate extract 2 g/100 mL having tannic acid before coloring and 1.5 g/100 mL of pomegranate extract after coloring silk has displayed colorfast shades of high strength.

The color strength of optimally dyeing and mordanted silk fabrics was analyzed. Table 4 shows fastness ratings demonstrating that the hues developed more quickly following chemical and bio-mordanting, monitored by dyeing under certain circumstances. This is because the MW radiation strengthened the colorant's ability to dye and brought it nearer to the fabric, as seen by making a solid bond between the fabric and the dye area. Bio-mordants have also enhanced the dye strength and fastness when techniques like rubbing, light washing, and washing are employed. Therefore, MW treatment enhanced the color fastness and color grading of Amba Haldi, a natural yellow color used in silk fabric coloring. In summary, bio-mordanting has been identified as a novel, cutting-edge method for making shade and improving the sustainability and environmental friendliness of fabric dyeing. These bio-mordant improve the durability of colored materials by allowing colorants to penetrate deeper into fibers or substrates and by catalyzing chemical alterations that strengthen the connections between dye molecules and the silk fabric. This enhanced resistance helps the dyed silk fabric withstand variables such as washing, light, and rubbing fastness^39^.

**Table 4:** Tonal variation and Colorfastness ratings of silk fabric pre and post-chemical and bio-mordanting at optimum conditions.

Mordant conc	K/S	L*	a*	b*	LF	WF	DRF	WRF
Al (pre 1.5%)	12.861	73.01	4.26	78.28	5	4/5	4/5	4/5
Al (post 1%)	10.443	75.34	3.47	77.62	5	4/5	4/5	4/5
Fe (pre 2%)	7.0705	55.17	8.07	34.88	5	4/5	4/5	4/5
Fe (post 1.5%)	13.879	56.20	11.86	52.70	5	4/5	4/5	4/5
TA (pre 1.5%)	17.426	59.71	8.59	62.31	5	4/5	4/5	4/5
TA (post 2%)	7.5166	56.22	6.29	36.68	5	4/5	4/5	4/5
Acacia (pre 1%)	10.205	70.57	2.97	61.86	5	4/5	4/5	4/5
Acacia (post 1.5%)	6.5272	66.46	0.40	47.74	5	4/5	4/5	4/5
Pomegranate (pre 2%)	10.899	75.54	4.18	68.91	5	4/5	4/5	4/5
Pomegranate (post 1.5%)	12.387	72.21	2.74	67.34	5	4/5	4/5	4/5
Pistachio (pre 1.5%)	6.9239	77.12	3.51	62.75	5	4/5	4/5	4/5
Pistachio (post 2%)	6.9704	72.62	0.35	56.42	5	4/5	4/5	4/5

LF: light fastness; WF: wash fastness; DRF: dry rubbing fastness; WRF: wet rubbing fastness.

## Conclusion

Microwave irradiation holds great potential for isolating natural colorants in mild environments, followed by silk fabric dyeing without compromising the colorant's physiological properties. MW-radiated silk fabric using a 7 pH extract, prepared from 4 g/100 mL of Amba Haldi powder at 80 °C for 45 min delivered excellent color strength, the experiment's results show decreased dyeing variables. In contrast to the natural yellow dyeing on radiated silk fabric, the best results were obtained, per the instructions, when the curcumin from Amba Haldi was isolated in an aqueous solution and MW-treated for 4 min. The significance of this cultural art has increased due to the sustainable method of the plant-based coloring of natural materials using bio-mordants. Hence, the utilization of the natural colors derived from Amba Haldi can serve as a viable and environmentally friendly alternative for coloring silk fabric in the textile dyeing and finishing industries.

## Data Availability

All data generated or analyzed during this study are included in this article.
